# Voriconazole is inferior to amphotericin B deoxycholate as the initial induction therapy for HIV-associated *Talaromyces marneffei* fungemia: A multicenter retrospective study

**DOI:** 10.1371/journal.pntd.0013012

**Published:** 2025-04-08

**Authors:** Shasha Ye, Jiaying Qin, Xingguo Miao, Guanjing Lang, Mengyan Wang, Gong Chen, Feifei Su, Lijun Xu

**Affiliations:** 1 National Clinical Research Center for Infectious Diseases, The First Affiliated Hospital, Zhejiang University School of Medicine, Hangzhou, China; 2 The State Key Laboratory for Diagnosis and Treatment of Infectious Diseases, The First Affiliated Hospital, Zhejiang University School of Medicine, Hangzhou, China; 3 Department of Infectious Diseases, Wenzhou Central Hospital, Wenzhou, Wenzhou, China; 4 Department II of Infectious Diseases, Xixi Hospital of Hangzhou, Zhejiang Chinese Medical University, Hangzhou, China; Duke University School of Medicine, UNITED STATES OF AMERICA

## Abstract

**Background:**

The optimal initial induction treatment for HIV-associated *Talaromyces marneffei* fungemia (HTMF) remains unclear.

**Methods:**

Three hundred and fifteen patients with HIV-associated *Talaromyces marneffei* were enrolled in this multicenter retrospective study. The effectiveness of induction regimens with amphotericin B deoxycholate (iAmBd), voriconazole (iVori), and a switch regimen from iVori to AmBd (iVori→AmBd switch) on 180-day all-cause mortality in HTMF patients was assessed.

**Results:**

The prevalence of HTMF was 75.9% (239/315) with a 180-day all-cause mortality of 20.1% (48/239). Among these HTMF patients, 44.4% (106/239) were treated with iAmBd, 41.8% (100/239) with iVori, and 13.8% (33/239) with other regimens. Additionally, 53% (53/100) of patients treated with iVori underwent an iVori→AmBd switch within 7 days. The 180-day cumulative survival rates were 88.7% for patients treated with iAmBd and 77.0% for those treated with iVori; 88.8% for patients who received AmBd within 3 days (iAmBd + 3-day iVori→AmBd switch) and 72.2% for those who continued iVori; 88.2% for patients who received AmBd within 5 days (iAmBd + 5-day iVori→AmBd switch) and 71.0% for those who continued iVori; 88.1% for those who received AmBd within 7 days (iAmBd + 7-day iVori→AmBd switch) and 66.0% for those who continued iVori (all log-rank P < 0.020). The prevalence of adverse drug reactions (ADRs) was 24.5% in the iAmBd group and 9.0% in the iVori group in induction stage (P < 0.001).

**Conclusion:**

Voriconazole is inferior to AmBd as an initial induction therapy for HTMF patients. Early AmBd administration or an early iVori→AmBd switch improves survival, despite the higher incidence of AmBd-related ADRs.

## Author summary

HIV-associated *Talaromyces marneffei* fungemia (HTMF) is a severe fungal infection with high mortality rates in people living with HIV (PLWH). Current guidelines recommend amphotericin B deoxycholate (AmBd) as the standard induction therapy. However, its use is often limited due to serious side effects such as kidney damage and electrolyte imbalances. Voriconazole, a newer antifungal drug, has been considered as an alternative due to its broader antifungal activity and fewer side effects. Our study, involving 315 patients across multiple centers, suggests that AmBd is superior to voriconazole in reducing 180-day mortality rates of HTMF. Early initiation of AmBd or early switch from voriconazole to AmBd significantly improved survival, despite the higher incidence of side effects. Our research highlights the need to balance drug efficacy with side effect management in treating severe *Talaromyces marneffei* infection.

## Introduction

*Talaromyces marneffei* (*T. marneffei*, TM) is a life-threatening opportunistic fungal infection prevalent among people living with HIV (PLWH) in Southeast Asia and Southern China [1–4]. TM primarily affects the monocyte-macrophage reticuloendothelial system, leading to either disseminated or localized infections [4–6]. HIV-associated *Talaromyces marneffei* fungemia (HTMF) is one of the most severe forms of this disease, with an in-hospital mortality rate of approximately 20% [7–9]. As a result, identifying the optimal antifungal regimen is crucial for improving patient outcomes.

Current guidelines recommend amphotericin B deoxycholate (AmBd) for 2 weeks as induction therapy, followed by itraconazole (400 mg daily for 10 weeks) as consolidation therapy, and then maintenance therapy with itraconazole (200 mg daily) until CD4 counts exceed 100 cells/mm³ for at least 6 months [10]. However, the use of AmBd is limited by its nephrotoxicity, leukopenia, and electrolyte disturbances [11,12]. Thus, there is a need to explore alternative treatments that can improve clinical outcomes.

Voriconazole (Vori) is a potent and broad-spectrum triazole antifungal agent with efficacy against a wide range of molds, yeasts, and dimorphic fungi [13–15]. Several studies have suggested that Vori is comparable to AmBd as induction therapy for HIV-associated *Talaromyces marneffei* (HTM) infection [16–18]. However, these studies based on small sample size often fail to distinguish between TM fungemia and localized TM infections. Furthermore, the bias as to who treated with AmBd and who treated with Vori as induction therapy is not fully addressed. Therefore, it is important to investigate whether AmBd and Vori are equally effective as initial induction therapies for HTMF. This multicenter retrospective study aims to compare the efficacy of AmBd versus Vori as initial induction therapies (iAmBd vs. iVori) in HTMF patients.

## Methods

### Ethics statement

This study protocol was conducted in accordance with the 1975 Declaration of Helsinki and was approved by the Ethics Committee of the First Affiliated Hospital of Zhejiang University School of Medicine (Hangzhou, China), Wenzhou Central Hospital (Wenzhou, China), Hangzhou Xixi Hospital (Hangzhou, China), and the Centers for Disease Control (CDC) (No. IIT20210532A). The ethical Committee waived the consent of patient. All data were analyzed anonymously.

### Study cohort and patient enrollment

Between January 2013 and August 2024, a total of 315 inpatient cases of HTM from the First Affiliated Hospital of Zhejiang University School of Medicine, Wenzhou Central Hospital, and Xixi Hospital of Hangzhou in China were eligible for inclusion in this study. Inclusion criteria were: a seropositive HIV result, age ≥18 years, and a diagnosis of Talaromycosis confirmed by culture, histopathology, or next-generation sequencing (NGS). Exclusion criteria were: seronegative HIV status, age <18 years, and absence of necessary laboratory and treatment records.

### Diagnosis criteria

HTM was diagnosed in PLWH who met at least one of the following criteria: (1)A positive culture result from clinical specimens such as blood, bone marrow, tissue biopsy, sputum, or body fluids; (2) Characteristic yeast cells (2–3 µm in diameter, with oval, round, or sausage-like shapes, and cells that divide by fission) observed in tissue sections by periodic acid-Schiff (PAS) or Wright’s staining; (3) Detection of *Talaromyces marneffei* DNA by NGS-based methods from blood, bronchoalveolar lavage fluid (BALF), body fluids, or tissue biopsies [10,19,20].

HTMF was defined as a positive peripheral blood culture or positive NGS result from blood in HTM patients exhibiting symptoms such as recurrent fever, rash, severe anemia, splenomegaly, and lymphadenopathy.

### Induction antifungal therapy

Antifungal therapy was initiated promptly upon diagnosis of Talaromycosis. The choice of induction regimen followed clinical guidelines and available antifungal agents [10,21], with therapy lasting at least 2 weeks.

### iAmBd regimen

Initial induction therapy with AmBd at a dose of 0.7–1.0 mg/kg/day via peripheral intravenous (IV) infusion.

### iVori regimen

Initial induction with voriconazole at 6 mg/kg IV every 12 hours for the first 24 hours, followed by 4 mg/kg IV daily.Empirical iVori was administered to patients with suspected Talaromycosis (characterized by fever, localized lymph node enlargement, low or nearly normal white blood cell (WBC) count, elevated C-reactive protein (CRP) levels, signs of fungal infection on pulmonary computed tomography (CT), and increased β-1,3-glucan in plasma) before the diagnosis was confirmed.

### iAmBd + iVori regimen

A combination of iAmBd and iVori, using the above dosages, was administered for at least 2 weeks as induction therapy.

### iVori→AmBd switch treatment

Treatment was switched from iVori to AmBd (iVori→AmBd switch) among those initially treated with iVori but switch to AmBd during induction treatment under the following conditions: (1) A confirmed diagnosis of HTM in patients previously suspected of Talaromycosis; (2) Intolerable iVori-related adverse drug reactions (ADRs); (3) Uncontrolled fever and worsening inflammatory markers, such as CRP or lactate dehydrogenase (LDH).

### Itraconazole regimen

In cases where AmBd or Vori were unavailable, oral itraconazole at 200 mg every 12 hours was used as the induction therapy.

### Consolidation therapy and maintenance therapy

After completing at least 14 days of initial induction therapy, all patients were given oral itraconazole at 400 mg daily for 10 weeks as consolidation therapy, followed by 200 mg daily for maintenance therapy (secondary prophylaxis) until their CD4 count remained above 100 cells/μL for more than 6 consecutive months.

### Outcome measures

The primary outcome was 180-day all-cause mortality. Secondary outcomes included the incidence of adverse drug reactions (ADRs) during induction treatment. ADRs that occurred after the induction period were not considered treatment-related. ADRs were documented in the electronic medical record system (EMRS) of each hospital and categorized according to the Common Terminology Criteria for Adverse Events (CTCAE) version 5.0 [22].

### Follow-up and data collection

Patient data, including demographics, laboratory results, clinical signs and symptoms, self-reported adherence, and regimen changes, were collected by hospital staff and local CDC personnel. This data was recorded in the electronic system of the National Free Antiretroviral Treatment Program (NFATP), which automatically generated an electronic database with data verification and cleaning capabilities [23]. Patients were followed from the first day of antifungal treatment until 180 days or death. The follow-up period ended on October 1, 2024. A total of 2.9% (9/315) of participants were lost to follow-up and were treated as right-censored data in the time-to-event analysis.

### Statistical analyses

Continuous variables were expressed as mean ± standard deviation for normally distributed data, and as median (interquartile range, IQR) for non-normally distributed data. Categorical variables were presented as frequency (percentage). Comparisons of continuous quantitative variables were made using the Student’s *t* test, Mann-Whitney *U* test, *χ* 2 analysis, or Fisher’s exact test. Data not available were defined as ‘missing data’. All-cause mortality was defined as an ‘event’. Data of patients were censored at the date of the final visit for those alive at the end of the follow-up period, at the date they were last known to be alive for those with unknown vital signs, or the date of participation for those wherein the cause of death was not known to be HTM-related. In the univariate and multivariate Cox regression analyses of risk factors of mortality, continuous variables were log_10_-transformed (log_10_) if they were not normally distributed. Covariates with P values < 0.050 in univariate analysis were further analyzed in the multivariate Cox proportional hazards model using the forward (likelihood ratio) method. Patient survival was analyzed by the Kaplan-Meier method, and the Log-rank test was used to assess significance. Tick marks on the KM curves represent censored data points, indicating patients who were lost to follow-up or event-free at the study cutoff. A P value <0.050 was considered statistically significant. Data analyses were performed using SPSS version 25.0 statistical software (IBM, Armonk, NY, USA) and GraphPad version 8.0 (GraphPad Software, La Jolla, California, USA).

## Results

### Demographic information and clinical characteristics of HTM patients

**Fig 1:**
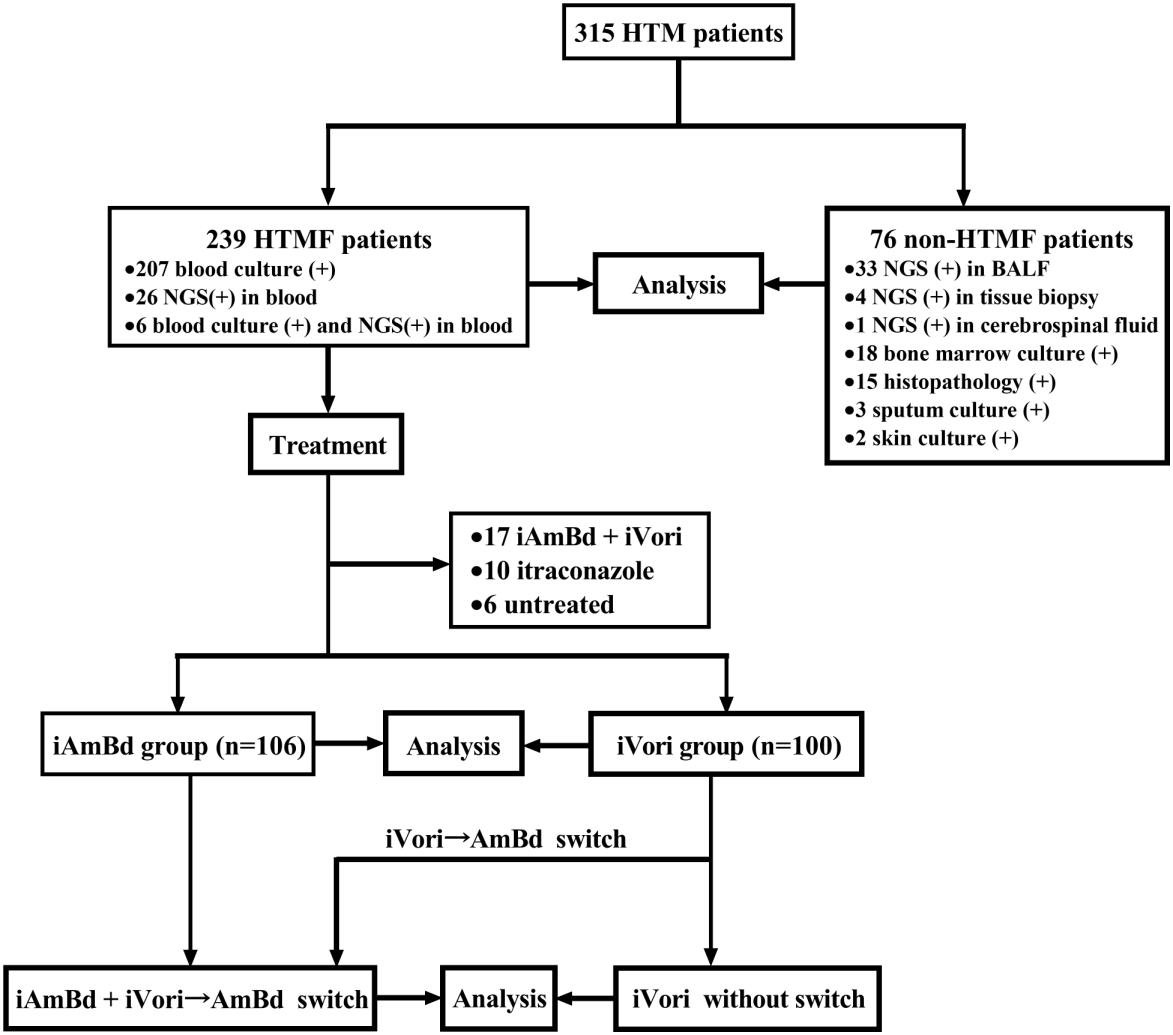
Study flowchart for patient selection. HTM, HIV-associated *Talaromyces marneffei*; HTMF, HIV-associated *Talaromyces marneffei* fungemia; (+), positive; NGS, next-generation sequencing; BALF, bronchoalveolar lavage fluid; iAmBd + iVori, a combination of iAmBd and iVori; iAmBd, initial induction treatment with amphotericin B deoxycholate; iVori, initial induction treatment with voriconazole; iVori→AmBd switch, switch regimen from initial treatment with voriconazole to AmBd during induction stage.

Among the 315 HTM patients enrolled in this study, 273 (86.7%) were male and 42 (13.3%) were female. The mean age was 38.9 ± 11.6 years, and the mean body mass index (BMI) was 19.9 ± 3.5 kg/m². Of the 315 patients, 239 (75.9%) HTM had fungemia (HTMF), and 76 (24.1%) did not. Among the HTMF patients, 207 (86.6%) were diagnosed via positive blood culture, 26 (10.9%) by positive blood NGS result, and 6 (2.5%) by both positive blood culture and NGS result. Of the 76 non-HTMF patients, diagnoses were made as follows: 33 (43.4%) by positive NGS result from BALF, 4 (5.3%) by NGS from tissue biopsy, 1 (1.3%) by NGS from cerebrospinal fluid, 18 (23.7%) by positive bone marrow culture, 15 (19.7%) by positive histopathology result, 3 (3.9%) by positive sputum culture, and 2 (2.6%) by positive skin culture. The patient selection flowchart was indicated in Fig 1.

There were no significant differences between HTMF and non-HTMF patients in terms of sex, age, BMI, comorbidities, or ART initiation. However, significant differences were observed in several laboratory parameters. HTMF patients had significantly lower levels of hemoglobin (g/L; 95.7 ± 19.2 vs. 102.5 ± 19.5, P = 0.008), platelets [× 10⁹/L; 94.0 (43.0-150.0) vs. 143.5 (93.3-217.0), P < 0.001], serum albumin (g/L; 28.2 ± 6.1 vs. 31.7 ± 5.0, P < 0.001), and CD4 count [cells/μL; 9.0 (4.0-20.0) vs. 20.0 (7.0-59.0), P < 0.001]. In contrast, HTMF patients had significantly higher levels of aspartate aminotransferase (AST) [U/L; 87.0 (45.0-155.0) vs. 37.0 (25.0-70.0), P < 0.001], CRP [mg/L; 64.7 (38.4-104.7) vs. 38.4 (9.65-80.0), P < 0.001], and LDH [U/L;470.0 (308.0-746.5) vs. 301.0 (202.3-455.5), P < 0.001] (Table 1).

**Table 1:** The baseline participant demographics, clinical, and laboratory features.

Factors	Overall (*N*=315)	All HTM patients (*N*=315)		P value	Overall (*N*=206)	Initial induction therapy (*N*=206)		P value
		HTMF(−) patients (*N*=76)	HTMF(+) patients (*N*=239)			iAmBd (*N*=106)	iVori (*N*=100)	
Sex (Male %)	273 (86.7)	64 (84.2)	209 (87.4)	0.470	181 (87.9)	95 (89.6)	86 (86.0)	0.426
Age (years)	38.9 ± 11.6	39.3 ± 11.9	38.8 ± 11.6	0.725	38.7 ± 11.4	38.7 ± 11.6	38.7 ± 11.3	0.998
Body mass index (kg/m^2^)	19.9 ± 3.5	20.0 ± 3.4	19.9 ± 3.6	0.941	20.0 ± 3.6	20.1 ± 3.4	19.8 ± 3.8	0.673
Predisposing diseases (PDs) [*n* (%)]								
Hypertension	12 (3.8)	4 (5.3)	8 (3.3)	0.677	7 (3.4)	3 (2.8)	4 (4.0)	0.937
Diabetes	9 (2.9)	4 (5.3)	5 (2.1)	0.294	5 (2.4)	1 (0.9)	4 (4.0)	0.331
Co-infection [*n* (%)]								
HBV infection	25 (7.9)	6 (7.9)	19 (7.9)	0.988	17 (8.3)	8 (7.5)	9 (9.0)	0.705
Syphilis	23(7.3)	7 (9.2)	16(6.7)	0.463	14 (6.8)	8 (7.5)	6 (6.0)	0.659
Tuberculosis	5 (1.6)	3 (3.9)	2 (0.8)	0.173	2 (1.0)	2 (1.9)	0 (0.0)	0.498
Non-tuberculous mycobacteria	9 (2.9)	3 (3.9)	6 (2.5)	0.795	6 (2.9)	5 (4.7)	1 (1.0)	0.242
Pneumocystis carinii pneumonia	43 (13.7)	13 (17.1)	30 (12.6)	0.314	26 (12.6)	12 (11.3)	14 (14.0)	0.563
Cryptococcosis	10 (3.2)	4 (5.3)	6 (2.5)	0.414	6 (2.9)	3 (2.8)	3 (3.0)	>0.999
Bacterial pneumonia	34 (10.8)	10 (13.2)	24 (10)	0.446	22 (10.7)	14 (13.2)	8 (8.0)	0.226
Cytomegalovirus	143 (45.4)	34 (44.7)	109 (45.6)	0.894	100 (48.5)	58 (54.7)	42 (42.0)	0.068
Epstein-Barr virus	124 (39.4)	33 (43.4)	91 (38.1)	0.406	73 (35.4)	39 (36.8)	34 (34.0)	0.675
Blood test								
WBC (× 10^9^/L)	3.3 (2.3-5.2)	3.3 (2.4-4.7)	3.4 (2.2-5.5)	0.986	3.5 (2.3-5.5)	3.5 (2.4-5.5)	3.6 (2.1-5.4)	0.821
Haemoglobin (g/L)	97.3 ± 19.5	102.5 ± 19.5	95.7 ± 19.2	0.008	95.2 ± 18.8	97.2 ± 18.3	93.0 ± 19.2	0.106
Platelets ( × 10^9^/L)	108.0 (52.0-177.5)	143.5 (93.3-217)	94.0 (43.0-150.0)^a^	<0.001	93.0 (43.0-159.0)	97.5 (55.3-167.3)^e^	86.0 (37.0-155.8)	0.141
Biochemical profile								
Serum albumin (g/L)	29.0 ± 6.0	31.7 ± 5.0	28.2 ± 6.1	<0.001	27.8 ± 5.8	28.8 ± 5.7	26.8 ± 5.7	0.011
ALT (U/L)	36.0 (21.8-67.3)	24.0 (14.3-44.3)	41.5 (24.0-70.0)^b^	<0.001	40 (24-68.5)	38 (22.8-69.0)	43 (26–68)^f^	0.546
AST (U/L)	71.0 (36.0-133.0)	37.0 (25.0-70.0)	87.0 (45.0-155.0)	<0.001	87.5 (44.8-160.3)	69.5 (36.5-118.0)	109.5 (51.3-207.5)	0.002
Total bilirubin (μmol/L)	9.4 (6.6-15.3)	6.9 (4.5-9.2)	10.9 (7.1-19.1)^b^	<0.001	11.0 (7.1-18.5)	9.8 (8.7-19.0)	11.9 (8.0-17.7)^f^	0.254
C-reactive protein (mg/L)	61.8 (30.5-101.7)	38.4 (9.65-80.0)	64.7 (38.4-104.7)^b^	<0.001	64.9 (39.9-105.4)	56.1 (30.8-107.6)^g^	73.5 (47.6-105.7)	0.077
Creatinine (μmol/L)	65.0 (56.0-78.0)	67.5 (56.0-78.0)	65 (56.0-78.0)	0.723	66.5 (55.8-79.0)	65.0 (55.0-77.3)	68.0 (56.0-84.0)	0.381
Creatinine clearance (ml/min)	103.7 (79.6-130.4)	104.0 (79.9-127.1)	103.7 (78.5-133.3)	0.673	103.5 (80.6-133.2)	105.1 (85.8-133.2)	98.7 (77.2-133.0)	0.375
Lactate dehydrogenase (U/L)	416.0 (277.0-662.3)	301.0 (202.3-455.5)	470.0 (308.0-746.5)^a^	<0.001	445.0 (301.0-726.0)	397.0 (274.3-542.8)^e^	537.0 (350.3-963.0)	<0.001
ART [*n* (%)]	84 (26.7)	24 (31.6)	60 (25.1)	0.266	51 (24.8)	30 (28.3)	21 (21.0)	0.225
CD4 (cells/μL)	10.0 (5.0-25.0)	20.0 (7.0-59.0)^c^	9.0 (4.0-20.0)^d^	<0.001	9.0 (4.0-19.3)	10.0 (5.0-22.0)^h^	8.0 (3.1-17.0)^f^	0.188
180-day overall morality [*n* (%)]	56 (17.8)	8 (10.5)	48 (20.1)	0.058	35 (17.0)	12 (11.3)	23 (23.0)	0.026
Survival days	22 (4-39)	44 (22-90)	16 (4-32)	0.041	20 (4-39)	42 (29-51)	5 (2-28)	0.002

Abbreviations: HTM, HIV-associated *Talaromyces marneffei*; HTMF, HIV-associated *Talaromyces marneffei* fungemia; HBV, Hepatitis B Virus; WBC, white blood cells; ALT, alanine aminotransferase; AST, aspartate aminotransferase; ART, antiretroviral therapy; iAmBd, initial induction treatment with amphotericin B deoxycholate; iVori, initial induction treatment with voriconazole.

^a^Data available in 237 patients; ^b^Data available in 238 patients; ^c^Data available in 73 patients; ^d^Data available in 235 patients;^e^Data available in 104 patients; ^f^Data available in 99 patients; ^g^Data available in 105 patients; ^h^Data available in 103 patients.

**Fig 2:**
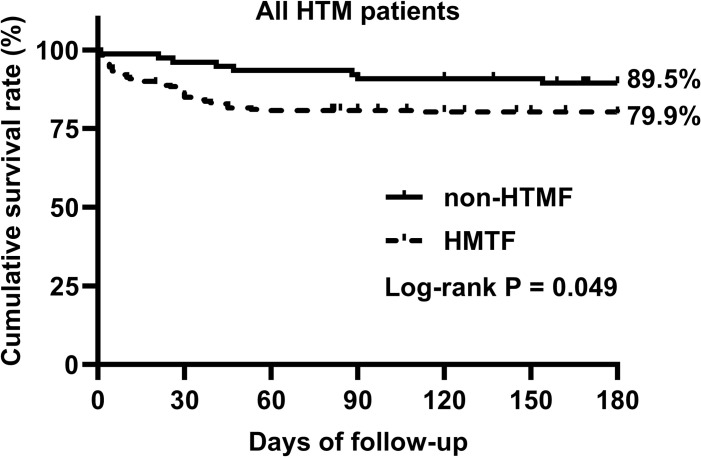
The 180-day cumulative survival rate in HTM patients with and without fungemia. HTMF, HIV-associated *Talaromyces marneffei* patient with fungemia; Non-HTMF, HIV-associated *Talaromyces marneffei* patient without fungemia.

During the 180-day follow-up, 56 (17.8%) HTM patients died, with a median survival time of 22 (4–39) days. Of these, 48 were HTMF patients and 8 were non-HTMF patients. The median survival time for HTMF patients who died was 16 (4–32) days, while the median survival time for non-HTMF patients who died was 44 (22–90) days (P = 0.041). The 180-day cumulative survival rate was 79.9% in HTMF patients and 89.5% in non-HTMF patients (Log-rank P = 0.049) (Fig 2). The demographic information and clinical characteristics of HTM patients are presented in Table 1.

### Comparison of 180-day all-cause mortality between iAmBd and iVori regimen in HTMF patients

**Fig 3:**
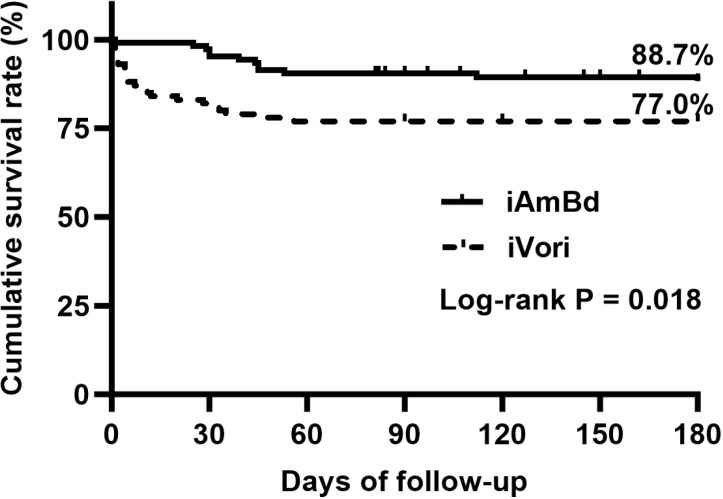
The 180-day cumulative survival rate in HTMF patients with different initial induction treatment. iAmBd, initial induction treatment with amphotericin B deoxycholate; iVori, initial induction treatment with voriconazle.

Among the 239 HTMF patients, 106 (44.3%) received iAmBd, 100 (41.8%) received iVori, 17 (7.1%) received combined iAmBd + iVori, 10 (4.2%) received itraconazole, and 6 (2.5%) abandoned treatment. After excluding patients who received itraconazole, combined iAmBd + iVori, or abandoned treatment due to small sample sizes, the remaining 206 HTMF patients were analyzed (Fig 1). Among these, 35 (17.0%) died during the 180-day follow-up, including 23 (11.2%) who died during hospitalization and 12 (5.8%) who died after discharge. The all-cause mortality was significantly lower in the iAmBd group (11.3%; 12/106) compared to the iVori group (23.0%; 23/100) (P = 0.026). The median survival time for patients who died in the iAmBd group was 42 (29–51) days, compared to 5 (2–28) days in the iVori group (P = 0.002). The 180-day cumulative survival rate was 88.7% for patients treated with iAmBd and 77.0% for those treated with iVori (Log-rank P = 0.018) (Fig 3).

### iVori treatment as an independent risk factor for 180-day all-cause mortality

There were no significant differences between the iVori and iAmBd groups in terms of sex, age, BMI, comorbidities, WBC count, hemoglobin, platelet count, CD4 count, or ART initiation. However, the serum albumin in iAmBd group was higher than that in iVori group [serum albumin (g/L): 28.8 ± 5.7 vs. 26.8 ± 5.7, P = 0.011], while levels of AST and LDH were significantly lower in the iAmBd group compared to the iVori group [AST (U/L): 69.5 (36.5-118.0) vs. 109.5 (51.3-207.5), P = 0.002; LDH (U/L): 397.0 (274.3-542.8) vs. 537.0 (350.3-963.0), P < 0.001] (Table 1).

A Cox proportional hazards model was used to analyze the effects of iAmBd and iVori on the 180-day cumulative survival rate after adjusting for serum albumin, AST, and LDH. In univariate analysis, elevated AST (log_10_) [HR: 2.646 (1.206-5.808); P = 0.015], creatinine (log_10_) [HR: 12.778 (2.623-62.237); P = 0.002], and LDH (log_10_) [HR: 3.725 (1.527-9.083); P = 0.004], as well as iVori treatment [HR: 2.259 (1.124-4.541); P = 0.022], were positively associated with 180-day all-cause mortality. Conversely, higher hemoglobin [HR: 0.977 (0.960-0.995); P = 0.011], platelet count (log_10_) [HR: 0.193 (0.084-0.443); P < 0.001], and serum albumin [HR: 0.884 (0.832-0.939); P < 0.001] were negatively associated with mortality. In multivariate analysis, elevated serum albumin [HR: 0.896 (0.842-0.953); P = 0.001] was negatively associated with 180-day mortality, while iVori treatment [HR: 2.189 (1.033-4.639); P = 0.041] was positively associated with mortality (S1 Data).

### iAmBd and early iVori→AmBd switch increased survival in HTMF patients

Among patients in the iVori group, 28.0% (28/100) underwent the iVori→AmBd switch regimen within 3 days, 10.0% (10/100) within 4–5 days, and 15.0% (15/100) within 6–7 days, and the remaining 47% (47/100) continued with iVori therapy. Totally, 28.0% (28/100) of patients underwent a 3-day iVori→AmBd switch, 38.0% (28 3day switch +10 4–5-day switch)) underwent a 5-day iVori→AmBd switch, and 53.0% (38 5-day switch+15 6–7-day switch) underwent a 7-day iVori→AmBd switch. Of those 53 patients who underwent iVori→AmBd switch, there were 36 underwent switch for confirmed HTM diagnosis from suspected Talaromycosis, 11 for uncontrolled fever, 4 for intolerable iVori-related ADRs, and 2 for increased CRP or LDH level. The indicators for regimen switching in each case are listed in S2 Data.

**Fig 4:**
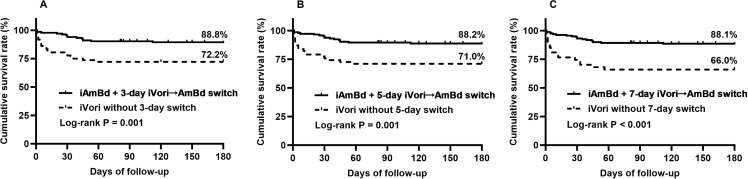
The 180-day cumulative survival rate in patients with different switch regimens. iAmBd, initial induction treatment with amphotericin B deoxycholate; iVori, initial induction treatment with voriconazle; iVori→AmBd switch, switch regimen from initial treatment with voriconazole to AmBd during induction stage.

**Fig 5:**
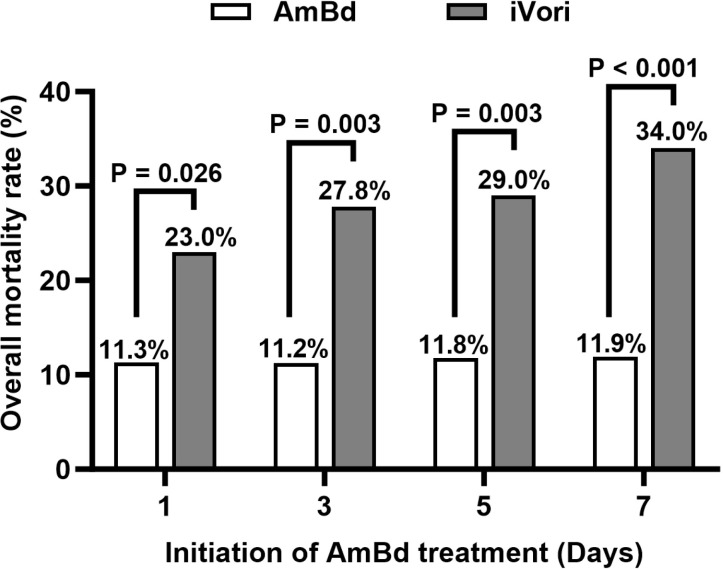
180-day all-cause mortality and initial AmBd treatment. AmBd: patients treated with iAmBd and switched AmBd; iVori: patients continued iVori without iVori→AmBd switch.

Overall, 134 patients received AmBd treatment within 3 days (106 iAmBd + 28 3-day iVori→AmBd switch), 144 patients received AmBd within 5 days (106 iAmBd + 38 5-day iVori→AmBd switch), and 159 patients received AmBd within 7 days (106 iAmBd + 53 7-day iVori→AmBd switch). The 180-day cumulative survival rates were 88.8% for patients treated with AmBd within 3 days, compared to 72.2% for those who continued iVori (Log-rank P = 0.001); 88.2% for patients treated with AmBd within 5 days versus 71.0% for those who continued iVori (Log-rank P = 0.001); and 88.1% for patients treated with AmBd within 7 days compared to 66.0% for those who continued iVori (Log-rank P < 0.001) (Fig 4A–C). The 180-day all-cause mortality was 11.3% for those treated with AmBd within 1 days and 23.0% for those treated with Vori without 1-day switch (P = 0.026), 11.2% for those treated with AmBd within 3 day and 27.8% for those treated Vori without 3-day switch (P = 0.003), and 11.8% for those treated with AmBd within 5 days and 29.0% for those treated with Vori without 5-day switch (P = 0.003), 11.9% for those treated with AmBd within 7 days and 34.0% for those treated with Vori without 7-day switch (P < 0.001), respectively (Fig 5).

### Adverse drug reactions

The overall incidence of ADRs during induction therapy was 24.5% (26/106) in the iAmBd group and 9.0% (9/100) in the iVori group (P < 0.001). In the iAmBd group, there were 34.6% (9/26) cases with creatinine elevation, 23.1% (6/26) with hypokalemia, 19.2% (5/26) with ALT elevation, 11.5% (3/26) with infusion-related reactions, 7.7% (2/26) with anemia and 3.8% (1/26) with gastrointestinal reactions. Of these, 88.5% (23/26) patients who experienced ADRs switched their treatment from AmBd to Vori, itraconazole, or posaconazole.

In the iVori group, the ADRs included 33.3% (3/9) ALT elevation, 22.2% (2/9) delirium, 11.1% (1/9) hallucination, 11.1% (1/9) had headache, 11.1% (1/9) skin allergy and 11.1% (1/9) visual abnormalities, respectively. Only 9.0% (9/100) of patients in the iVori group switched to iVori→AmBd due to intolerable ADRs.

Furthermore, among those HTMF patients who underwent iVori→AmBd switch, 17.0% (9/53) experienced new AmBd-related ADRs. Detailed information on ADRs is presented in S3 Data.

## Disscussion

The optimal initial induction treatment for HTMF remains unclear. Our study provides several important findings: (1) The prevalence of HTMF was 75.9%; (2) the 180-day all-cause mortality rate for HTMF patients was 20.1%, higher than the 10.5% seen in non-HTMF patients; (3) although the overall incidence of ADRs was significantly higher in the iAmBd group compared to the iVori group, the iAmBd regimen was associated with improved survival rates, whereas delayed AmBd treatment significantly increased the mortality in HTMF patients.

HTMF is a life-threatening infection in PLWH [24–26]. In our study, we found that HTMF patients had significantly lower levels of hemoglobin, platelets, serum albumin, and CD4 count, while exhibiting higher levels of AST, CRP, and LDH, compared to non-HTMF patients. These findings suggest that HTMF patients are in a severely deteriorated health condition with a high burden of organ dysfunction, which increases the risk of mortality. Despite prompt antifungal therapy being administered to most HTMF patients, the mortality rate remained high at 20.1%. Therefore, early and potent antifungal regimens are critical to improving clinical outcomes for HTMF patients.

AmBd continues to be the first-line and standard treatment [27]. However, its use is limited by a range of adverse effects, including infusion-related reactions, hypokalemia, anemia, gastrointestinal issues, hepatotoxicity, and nephrotoxicity [28]. This highlights the urgent need for effective and safer alternative antifungal therapies. Voriconazole (Vori), a triazole with broad-spectrum antifungal activity, has shown efficacy against *Talaromyces marneffei* in both in vitro and in vivo studies [16,17,29]. One study demonstrated that Vori as induction therapy was comparable to AmBd in terms of therapeutic efficacy over a 48-week period in HTM patients [18]. However, another study reported that Vori exhibited a higher minimum inhibitory concentration (MIC) for *T. marneffei*, leading to delayed clearance of the pathogen from the bloodstream [30]. We hypothesize that the heterogeneity of fungal infections (fungemia versus localized infection) was not adequately addressed in these studies, potentially masking the detrimental impact of fungemia on mortality. In our study, we found that iVori treatment was associated with a 2.2-fold higher risk of mortality compared to iAmBd in HTMF patients after adjusting for confounding factors. Notably, patients who received iAmBd treatment or underwent an early iVori→AmBd switch had higher survival rates compared to those who continued iVori treatment. Therefore, early initiation of AmBd treatment is crucial for improving the prognosis of HTMF patients.

The superiority of iAmBd over iVori in our study may be explained by the following factors: (1) **Mechanism of Action**: AmBd, a polyene antifungal, exerts a potent fungicidal effect by binding to ergosterol, a component of fungal cell membranes, leading to pore formation and subsequent cell death [31]. In contrast, voriconazole is a fungistatic agent, which may be less effective in cases of high fungal burden or severe infection [13,32]. (2) **Pharmacokinetics**: *T. marneffei* primarily infects the monocyte-macrophage reticuloendothelial system, facilitating its spread to organs such as the lungs, liver, and spleen [19]. AmBd can achieve high concentrations and long-lasting activity in these organs [33], making it more effective in treating disseminated infections like HTMF.

Our study has some limitations. First, it is a retrospective cohort study, which may introduce biases. Our data also suggest that physicians may prefer Vori over AmBd as the initial induction treatment for critically ill HTMF patients to avoid the toxicity associated with AmBd. Notably, patients in the iVori group had significantly lower serum albumin levels and higher AST and LDH levels, reflecting a worse health status or greater organ injury. Although our analysis showed that iVori treatment remained an independent risk factor for higher 180-day all-cause mortality after adjusting for hypoalbuminemia, AST, and LDH in the multivariate model, a randomized controlled trial (RCT) is need clarify the efficacy on AmBd vs Vori on HTMF treatment. Second, we only considered AmBd for the treatment of HTMF and did not include liposomal amphotericin B due to its unavailability in China. Third, during the induction treatment period, blood levels of Vori were not monitored in patients receiving iVori therapy. Consequently, it is possible that suboptimal iVori levels contributed to its inferior effectiveness compared to iAmBd.

In conclusion, voriconazole is inferior to AmBd as the initial induction therapy for HTMF patients. Early AmBd administration and early iVori→AmBd switch are significantly associated with improved survival rates, despite the higher incidence of AmBd-related ADRs. A prospective study is urgently needed to compare the efficacy of AmBd versus Vori as induction therapy, with the goal of refining current guidelines for HTM and HTMF treatment.

## Supporting information

S1 Data Risk factors in the multivariate Cox proportional hazards model. (DOCX)

S2 Data Detailed reasons for switching from iVori to AmBd (iVori→AmBd switch). (DOCX)

S3 Data ADRs between the iAmBd group and iVori group. iAmBd, initial induction treatment with amphotericin B deoxycholate; iVori, initial induction treatment with voriconazole; iVori→AmBd switch, switch regimen from initial treatment with voriconazole to AmBd during induction stage; ADRs, adverse drug reactions; →, switch to other drugs; ALT, alanine aminotransferase.
(TIF)

S4 Data The original data for statistic in our study. (XLSX)
