# Supplementary material for: Voriconazole is inferior to amphotericin B deoxycholate as the initial induction therapy for HIV-associated Talaromyces marneffei fungemia: A multicenter retrospective study
Source: PLoS Negl Trop Dis. 2025 Apr 8;19(4):e0013012. doi: 10.1371/journal.pntd.0013012 (PMC12121904; doi:10.1371/journal.pntd.0013012)
Supplement: S1 Data — (DOCX) [file pntd.0013012.s001.docx]

Risk factors for mortality in HTMF patients: univariate and multivariate Cox proportional hazard models (*N*=206)

|  |  | **Risk factors for 180-day mortality** | | | | |
| --- | --- | --- | --- | --- | --- | --- |
|  |  | **univariate** | |  | **multivariate** | |
| **Factors** | **Deaths (*N*=35)** | **HR (95% CI)** | **P Value** |  | **HR (95% CI)** | **P Value** |
| Sex |  |  |  |  |  |  |
| Male | 29/181 | 1 |  |  |  |  |
| Female | 6/25 | 1.530 (0.635-3.686) | 0.343 |  |  | - |
| Age (years) | - | 1.027 (0.999-1.056) | 0.062 |  |  | - |
| Body mass index (kg/m^2^) | - | 0.948 (0.857-1.049) | 0.301 |  |  | - |
| Predisposing diseases (PDs) |  |  |  |  |  |  |
| Yes | 2/11 | 1 |  |  |  |  |
| No | 33/195 | 0.905 (0.217-3.773) | 0.891 |  |  | - |
| Co-infection |  |  |  |  |  |  |
| Yes | 27/156 | 1 |  |  |  |  |
| No | 8/50 | 0.933 (0.424-2.054) | 0.864 |  |  | - |
| WBC (×109 /L) (log_10_) | - | 2.597 (0.745-9.056) | 0.134 |  |  | - |
| Hemoglobin (g/L) | - | 0.977 (0.960-0.995) | 0.011 |  |  | - |
| Platelets ( × 10^9^ /L) (log_10_)^a^ | - | 0.193 (0.084-0.443) | <0.001 |  |  | - |
| Serum albumin (g/L) | - | 0.884 (0.832-0.939) | <0.001 |  | 0.896 (0.842-0.953) | 0.001 |
| AST (U/L) (log_10_) | - | 2.646 (1.206-5.808) | 0.015 |  |  | - |
| Creatinine (μmol/L) (log_10_) | - | 12.778 (2.623-62.237) | 0.002 |  |  | - |
| CRP (mg/L) (log_10_)^b^ | - | 1.637 (0.936-2.861) | 0.084 |  |  | - |
| LDH (U/L) (log_10_)^a^ | - | 3.725 (1.527-9.083) | 0.004 |  |  | - |
| CD4 count (cells/μL) (log_10_)^c^ | - | 1.008 (0.525-1.933) | 0.982 |  |  | - |
| ART |  |  |  |  |  |  |
| On | 8/51 | 1 |  |  |  |  |
| Off | 27/155 | 1.131 (0.514-2.489) | 0.761 |  |  | - |
| Initial induction therapy |  |  |  |  |  |  |
| iAmBd | 12/106 | 1 |  |  | 1 |  |
| iVori | 23/100 | 2.259 (1.124-4.541) | 0.022 |  | 2.189 (1.033-4.639) | 0.041 |

Log_10_: log_10_-transformation to adjust for non-normal distributed data

Abbreviations: HTMF, HIV-associated *Talaromyces marneffei* fungemia; WBC, white blood cell; AST, alanine aminotransferase; CRP, C-reactive protein; LDH, lactate dehydrogenase; ART, antiretroviral therapy; iAmBd, initial induction with amphotericin B deoxycholate; iVori, initial induction with voriconazole

^a^Data available in 204 patients

^b^Data available in 205 patients

^c^Data available in 202 patients
