# Supplementary material for: Voriconazole is inferior to amphotericin B deoxycholate as the initial induction therapy for HIV-associated Talaromyces marneffei fungemia: A multicenter retrospective study
Source: PLoS Negl Trop Dis. 2025 Apr 8;19(4):e0013012. doi: 10.1371/journal.pntd.0013012 (PMC12121904; doi:10.1371/journal.pntd.0013012)
Supplement: S2 Data — (DOCX) [file pntd.0013012.s002.docx]

**Supplement 2** Reasons for switching from iVori to AmBd (iVori→AmBd switch)

| **Case** | **Reason** |
| --- | --- |
| **3-day iVori-AmBd switch (*N*=28)** |  |
| Case 4 | intolerable iVori-related ADRs (skin allergy) |
| Case 7 | confirmed HTM diagnosis (blood culture) |
| Case 17 | confirmed HTM diagnosis (blood culture) |
| Case 30 | confirmed HTM diagnosis (blood culture) |
| Case 45 | confirmed HTM diagnosis (blood culture) |
| Case 46 | confirmed HTM diagnosis (blood culture) |
| Case 56 | confirmed HTM diagnosis (blood culture) |
| Case 69 | uncontrolled fever |
| Case 70 | confirmed HTM diagnosis (blood culture) |
| Case 71 | confirmed HTM diagnosis (blood culture) |
| Case 72 | uncontrolled fever |
| Case 88 | confirmed HTM diagnosis (blood culture) |
| Case 98 | confirmed HTM diagnosis (blood NGS positive) |
| Case 109 | confirmed HTM diagnosis (blood culture) |
| Case 128 | uncontrolled fever |
| Case 130 | confirmed HTM diagnosis (blood culture) |
| Case 134 | confirmed HTM diagnosis (blood NGS positive) |
| Case 217 | uncontrolled fever |
| Case 218 | intolerable iVori-related ADRs (hallucination) |
| Case 242 | uncontrolled fever |
| Case 260 | deteriorated inflammatory indicators (CRP and LDH elevation) |
| Case 265 | intolerable iVori-related ADRs (visual abnormalities) |
| Case 268 | intolerable iVori-related ADRs (ALT elevation) |
| Case 278 | confirmed HTM diagnosis (blood culture) |
| Case 286 | deteriorated inflammatory indicators (CRP elevation) |
| Case 297 | confirmed HTM diagnosis (blood culture) |
| Case 300 | confirmed HTM diagnosis (blood culture) |
| Case 315 | confirmed HTM diagnosis (blood culture) |
| **4-5day iVori-AmBd switch (*N*=10)** |  |
| Case 36 | confirmed HTM diagnosis (blood culture) |
| Case 113 | confirmed HTM diagnosis (blood culture) |
| Case 133 | confirmed HTM diagnosis (blood culture) |
| Case 140 | confirmed HTM diagnosis (blood NGS positive) |
| Case 184 | confirmed HTM diagnosis (blood culture) |
| Case 216 | uncontrolled fever |
| Case 229 | uncontrolled fever |
| Case 275 | confirmed HTM diagnosis (blood culture) |
| Case 294 | confirmed HTM diagnosis (blood culture) |
| Case 305 | uncontrolled fever |
| **6-7day iVori-AmBd switch (*N*=15)** |  |
| Case 19 | confirmed HTM diagnosis (blood culture) |
| Case 44 | confirmed HTM diagnosis (blood culture) |
| Case 52 | confirmed HTM diagnosis (blood culture) |
| Case 99 | confirmed HTM diagnosis (blood NGS positive) |
| Case 107 | confirmed HTM diagnosis (blood culture) |
| Case 215 | confirmed HTM diagnosis (blood culture) |
| Case 228 | uncontrolled fever |
| Case 237 | confirmed HTM diagnosis (blood culture) |
| Case 259 | uncontrolled fever |
| Case 270 | confirmed HTM diagnosis (blood culture) |
| Case 272 | confirmed HTM diagnosis (blood culture) |
| Case 276 | confirmed HTM diagnosis (blood culture) |
| Case 293 | confirmed HTM diagnosis (blood culture) |
| Case 301 | confirmed HTM diagnosis (blood culture) |
| Case 313 | uncontrolled fever |

Abbreviations: iVori, initial induction with voriconazole; AmBd, amphotericin B deoxycholate; ADRs, adverse drug reactions; HTM, HIV-associated *Talaromyces marneffei*; NGS, next-generation sequencing; CRP, C-reactive protein; LDH, lactate dehydrogenase; ALT, alanine aminotransferase.
