# Supplementary figures and images for: Voriconazole is inferior to amphotericin B deoxycholate as the initial induction therapy for HIV-associated Talaromyces marneffei fungemia: A multicenter retrospective study
Source: PLoS Negl Trop Dis. 2025 Apr 8;19(4):e0013012. doi: 10.1371/journal.pntd.0013012 (PMC12121904; doi:10.1371/journal.pntd.0013012)

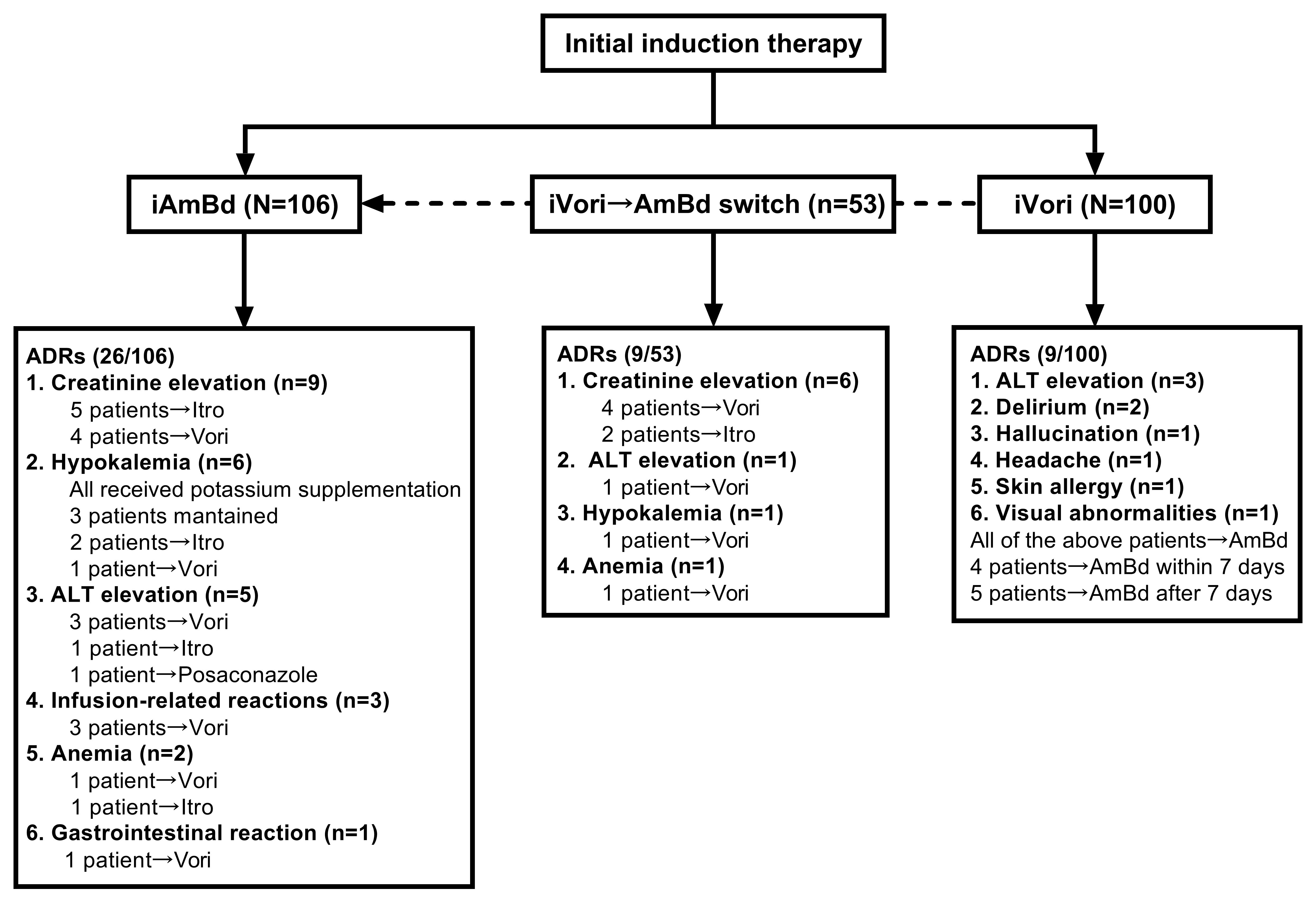

Supplement: S3 Data — iAmBd, initial induction treatment with amphotericin B deoxycholate; iVori, initial induction treatment with voriconazole; iVori→AmBd switch, switch regimen from initial treatment with voriconazole to AmBd during induction stage; ADRs, adverse drug reactions; →, switch to other drugs; ALT, alanine aminotransferase. (TIF) [file pntd.0013012.s003.tif]
